# The preventive effect of dexmedetomidine on anesthesia complications in strabismus surgery: a systematic review and meta-analysis

**DOI:** 10.1186/s12871-023-02215-9

**Published:** 2023-07-25

**Authors:** Yiren Chen, Mingjie Li, Yajing Zheng, Ailuan Chen, Chengjie Li

**Affiliations:** 1 Department of Anesthesiology, Hainan Eye Hospital and Key Laboratory of Ophthalmology, Zhongshan Ophthalmic Center, Sun Yat-Sen University, Haikou, 570311 China; 2 Department of Anesthesiology, Chengmai County People’s Hospital, Chengmai, 571900 China; 3 Department of Ophthalmology, Hainan Eye Hospital and Key Laboratory of Ophthalmology, Zhongshan Ophthalmic Center, Sun Yat-Sen University, Haikou, 570311 China; 4 Department of Anesthesiology, Hainan Western Central Hospital, Danzhou, 571700 Hainan China

**Keywords:** Dexmedetomidine, Strabismus surgery, Anesthesia complications, Meta-analysis

## Abstract

**Objective:**

Dexmedetomidine is a medication that has analgesic, sedative, and anti-anxiety properties. In the clinical, it is often used to prevent common complications associated with strabismus surgery, including postoperative delirium, postoperative nausea and vomiting, postoperative pain, and oculocardiac reflex. However, its effectiveness and side effects of the present studies are different. The sample sizes of the present studies on the prevention of complications of dexmedetomidine are small. Therefore, this study evaluates the efficacy of dexmedetomidine in preventing anesthesia-related complications in strabismus surgery through a systematic review and meta-analysis.

**Methods:**

Literature was retrieved from 10 commonly used databases and randomized controlled trials published up to May 2022 were sought. The included studies compared the intervention effects of dexmedetomidine versus placebo on anesthesia-related complications in surgery. The occurrence rates of postoperative delirium, postoperative nausea and vomiting, postoperative pain, and oculocardiac reflex in patients undergoing strabismus surgery were evaluated. Statistical analyses and forest plots were generated using Review Manager and STATA software. Binary outcomes were measured using relative risk (RR) with a 95% confidence interval for each outcome. The Cochrane risk of bias tool was used to assess the bias and risk in the studies that met the inclusion criteria.

**Results:**

A total of 13 articles were ultimately included in the analysis, comprising 1,018 patients who underwent strabismus surgery. The dexmedetomidine group, compared to the placebo group, demonstrated significant reductions in the incidence of postoperative delirium (RR = 0.73, *P* = 0.001), severe postoperative delirium (RR = 0.45, *P* = 0.005), postoperative nausea and vomiting (RR = 0.48, *P* < 0.0001), and the need for supplemental analgesia postoperatively (RR = 0.60, *P* = 0.004). Additionally, subgroup analysis revealed that intravenous administration of dexmedetomidine significantly reduced the incidence of oculocardiac reflex (RR = 0.50, *P* = 0.001). In contrast, intranasal administration of dexmedetomidine did not have a significant effect on the incidence of oculocardiac reflex (RR = 1.22, *P* = 0.15). There was a significant difference between the subgroups (*P* = 0.0005, I2 = 91.7%).

**Conclusion:**

Among patients undergoing strabismus surgery, the use of dexmedetomidine can alleviate postoperative delirium and reduce the incidence of postoperative nausea and vomiting, as well as postoperative pain. Moreover, intravenous administration of dexmedetomidine can lower the occurrence rate of the oculocardiac reflex.

**Supplementary Information:**

The online version contains supplementary material available at 10.1186/s12871-023-02215-9.

## Introduction

Strabismus surgery is one of the common procedures in ophthalmology, often performed under general anesthesia [1]. Patients frequently experience postoperative delirium, postoperative nausea and vomiting (PONV), and postoperative pain following strabismus surgery. According to the literature, the incidence rates of these complications can be as high as 40%-84%, 37%-80%, and 65% respectively [2]. Postoperative delirium can even lead to patients removing intravenous catheters or causing self-harm [3]. Severe postoperative vomiting can affect patients’ ability to eat and drink, potentially leading to electrolyte imbalances, which in turn can hinder recovery and result in prolonged hospital stays or unplanned readmissions [4]. Postoperative pain can also affect oral intake, sleep, and may prolong hospitalization [4]. Hence, complications following strabismus surgery not only increase patients' distress and discomfort but also add to the workload and stress of medical or nursing staff [2, 4].

Dexmedetomidine is a highly selective α2-adrenoceptor agonist, known for its sedative, analgesic, and anti-anxiety effects, making it widely used in clinical anesthesia [5]. Dexmedetomidine can be used for the prevention or treatment of delirium in intensive care units [6, 7], and can also be used to prevent postoperative delirium in adults undergoing cardiac or other non-cardiac surgeries [8]. Furthermore, literature reports that dexmedetomidine can alleviate pain and delirium in children undergoing adenoidectomy without significant side effects [9]. Moreover, a meta-analysis reported that the use of dexmedetomidine reduced the incidence of postoperative nausea and vomiting in children undergoing various types of surgery [10]. However, there are limited and somewhat outdated meta-analyses on whether dexmedetomidine can reduce the incidence of postoperative delirium, nausea, vomiting, and pain in patients undergoing strabismus surgery. Additionally, as intranasal administration of dexmedetomidine becomes increasingly utilized in clinical settings, literature analyzing this method of administration remains scarce. Also, existing studies often have small sample sizes, and the results are frequently contradictory [11, 12], which is confusing. Therefore, there is still a need for high-quality meta-analyses to evaluate the efficacy of dexmedetomidine in preventing anesthesia-related complications in strabismus surgery.

In this study, we conducted a comprehensive and systematic review and meta-analysis of randomized controlled trials (RCTs) concerning dexmedetomidine and strabismus surgery, to assess the efficacy of dexmedetomidine in preventing anesthesia-related complications in strabismus surgery.

## Materials and methods

### Search strategy and inclusion criteria

The report of this article was followed the Preferred Reporting Items for Systematic Reviews and Meta-Analyses (PRISMA) statement guidelines [13]. The protocol of this systematic review was registered in the International prospective register of systematic reviews (PROSPERO), National Institute for Health research (ID: CRD42023438847). In adherence to the principles of Cochrane, three researchers independently searched PubMed, Medline, Embase, Ovid, Cochrane Library, ISI Web of Science, Clinical Trials, and Chinese databases, including China National Knowledge Infrastructure (CNKI), Wanfang Database, and Chinese Biomedical Literature Database (SinoMed), to retrieve eligible studies published up until May 2022 since the inception of these databases. Keywords used in the search included "dexmedetomidine," "α2 agonist," "children," "strabismus," "eye surgery," and "ophthalmic surgery." After generating a list of potentially eligible studies, the researchers conducted manual review and verification to determine the final selection for inclusion in the analysis.

This study only included Randomized Controlled Trials (RCTs) related to strabismus surgery that compared the use of dexmedetomidine with a placebo or other anesthetic agents, without restrictions on the route of administration or dosage of the drug. Studies that were of types such as reviews, cohort studies, or case reports, and other non-RCT studies were excluded. We only included the studies written in English or Chinese. The three independent researchers then screened the retrieved literature, removed duplicates that arose during database integration, and read the titles and abstracts of the articles for initial selection. The full texts of the preliminarily selected articles were obtained and thoroughly read to determine if they met the inclusion criteria. Any discrepancies during the process were resolved through consultation and discussion.

The primary outcomes of interest in this study were the incidence of postoperative delirium, postoperative nausea and vomiting (PONV), and postoperative pain as well as the number of patients requiring additional analgesics postoperatively. Additionally, the study also assessed the incidence of the oculocardiac reflex (OCR).

### Data extraction

Two independent researchers extracted the necessary information from the included studies, including author, publication year, patient recruitment period, inclusion criteria, sample size, baseline data, comparison groups, intervention duration, primary anesthetic agents, and data on primary and secondary outcomes. A third researcher was involved in information verification and data cross-checking to ensure the accuracy and reliability of the information and data.

### Literature quality assessment

Two researchers assessed the bias and risk of the eligible studies using the Cochrane Risk of Bias tool [14]. This tool includes the following sections: selection bias due to random sequence generation or allocation concealment; performance bias due to participants or personnel being aware of the assigned interventions; detection bias due to blinding deficiencies when obtaining outcomes; reporting bias due to selective reporting of outcomes, and other biases. Each risk of bias level is categorized as low, high, or unclear, and the results are represented with different colored blocks and corresponding risk of bias graphs. In case of discrepancies in the evaluation, the assessment of a third researcher is adopted for the final evaluation.

### Statistical analysis

For dichotomous variables, this study employs the risk ratio (RR) along with the corresponding 95% confidence intervals (CI) for outcome analysis. The heterogeneity of the studies is assessed using the I^2^ statistic and the chi-square test, and if substantial heterogeneity is observed, subgroup analyses are conducted to explore the reasons. I^2^ value is more than 50%, which means the high heterogeneity and the random model should be used. I^2^ value is less than 50%, which means the low heterogeneity and fixed model should be used. Egger's test are employed to assess publication bias. Forest plots and funnel plots are created using Review Manager software (Version 5.4, The Nordic Cochrane Centre, Copenhagen, Denmark). Statistical analyses are performed using STATA (Version 14.0, Stata Corp, College Station, TX, USA). A difference is considered to be statistically significant when *P* < 0.05.

## Results

### Search results and literature quality evaluation

**Fig. 1:**
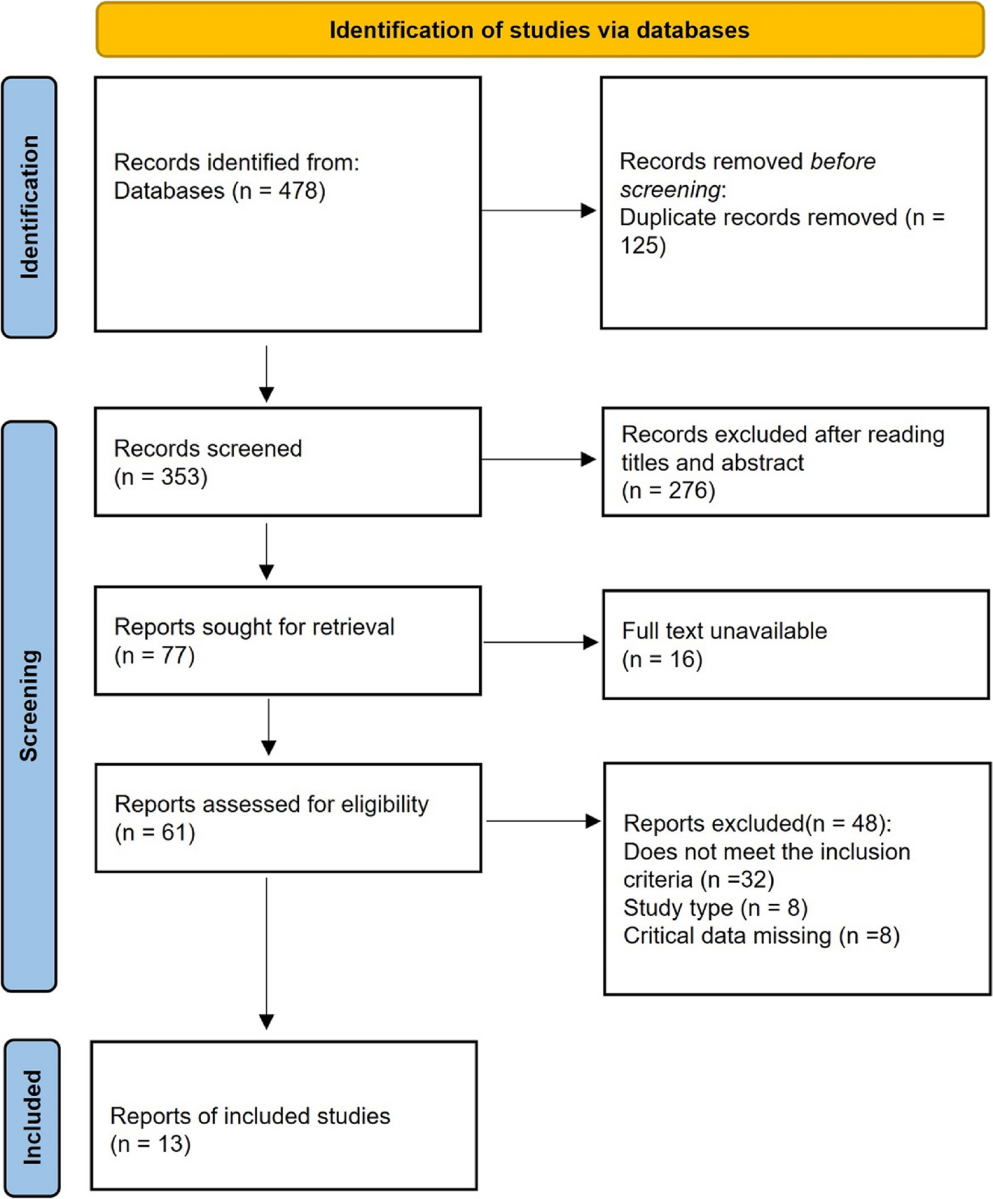
Flowchart of literature screening

Figure 1 depicts the flow of study selection and screening. In this research, a comprehensive and exhaustive search was carried out across common databases, yielding a total of 478 search results. After merging the databases, 125 duplicate records were removed. An initial screening was conducted by reviewing titles and abstracts, resulting in the exclusion of 276 articles, along with the removal of 16 articles for which the full text was not accessible. This left a total of 61 articles. All 61 articles underwent detailed full-text review and examination. Subsequently, 32 articles that did not meet the inclusion criteria, 8 non-randomized controlled trial (RCT) articles, and 8 articles where key data needed for the study were not obtainable were excluded. Ultimately, 13 studies that fulfilled the research criteria were included in the analysis for further quantitative and qualitative assessments [11, 12, 15–25]. Of these, 4 studies utilized intranasal administration [11, 12, 18, 23], while the remaining 9 employed intravenous administration [15–17, 19–22, 24, 25]. The characteristics of the included studies were shown in the Table 1.

**Table 1:** Summary of the included studies

First author	Publication year	Data source	Type of study	number of cases (experimental group/control group)	Total number of cases (n)	Method of administration
Wu SR [11]	2020	China	RCT	45/45	90	Intranasal
Chu L [12]	2021	China	RCT	70/70	140	Intranasal
Chen JY [14]	2013	China	RCT	28/28	56	Intravenous
Ye WD [15]	2014	China	RCT	30/30	60	Intravenous
Kim J [16]	2014	Korea	RCT	47/47	94	Intravenous
Abdelaziz HMM [17]	2016	Egypt	RCT	35/35	70	Intranasal
Song IA [18]	2016	Korea	RCT	25/25	50	Intravenous
Abdel-Rahman KA [19]	2018	Egypt	RCT	30/30	60	Intravenous
Dai BZ [20]	2020	China	RCT	48/48	96	Intravenous
Li S [21]	2020	China	RCT	42/42	84	Intravenous
Yao Y [22]	2020	China	RCT	32/32	64	Intranasal
Elghamry MR [23]	2021	Egypt	RCT	35/35	70	Intravenous
Oriby ME [24]	2021	Qatar	RCT	42/42	84	Intravenous

### Risk of bias assessment

**Fig. 2:**
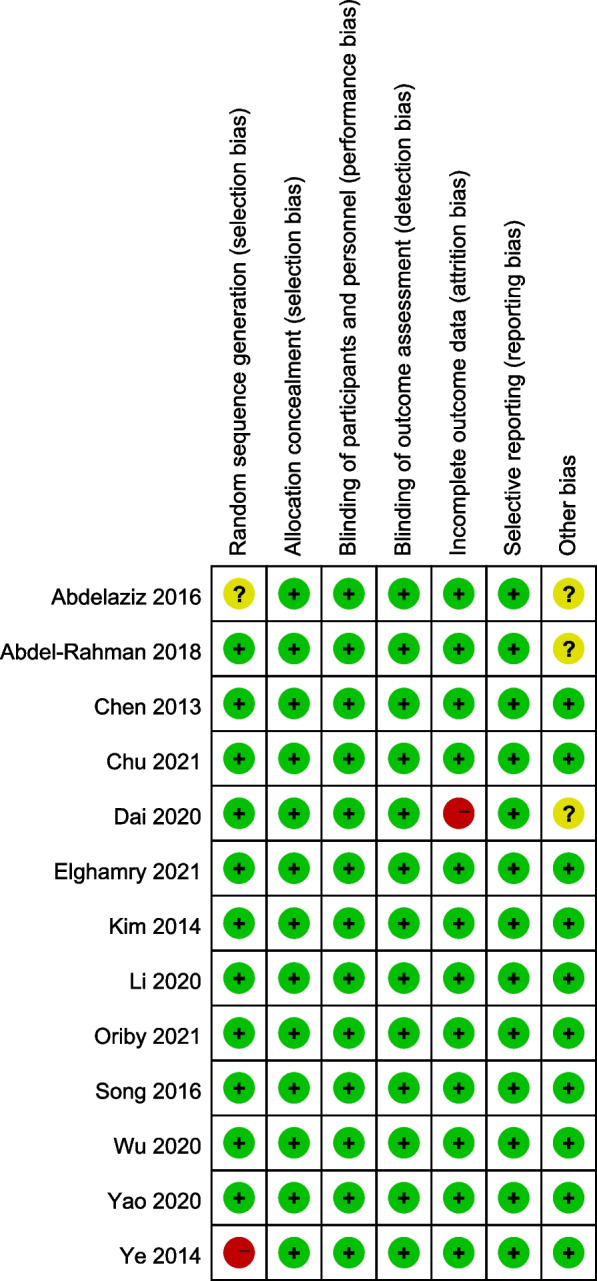
Assessment of risk of methodological bias in the included studies

Figure 2 displays the results of the bias risk assessment. The majority of studies had a low risk of bias, with two instances containing high-risk bias scores: one study did not employ blinding during random allocation [16], and another study exhibited high risk during outcome evaluation [21]. Additionally, three studies were considered to have unclear other risks because the sample size was not calculated during the trial design [18, 20, 21].

### Postoperative delirium

**Fig. 3:**
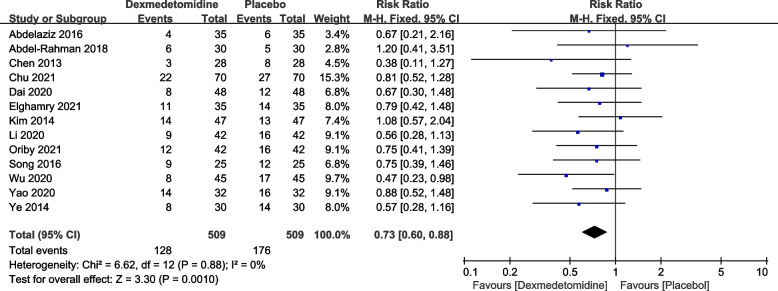
Forest plot of postoperative delirium incidence DEX, dexmedetomidine

This study analyzed the incidence of postoperative delirium and severe delirium with dexmedetomidine compared to a placebo (saline solution). A total of 13 articles reported the number of cases of postoperative delirium, and no significant heterogeneity was found among the studies (*P* = 0.88, I2 = 0%). A fixed-effects model was utilized. The pooled RR value was 0.73, with a 95% CI of (0.60, 0.88), and the combined effect size Z = 3.30 (*P* = 0.001). The results showed that compared to the placebo group, the dexmedetomidine group had a significantly lower incidence of postoperative delirium, as seen in Fig. 3. Egger's tests (*P* = 0.0085) indicated that there was no publication bias.

**Fig. 4:**
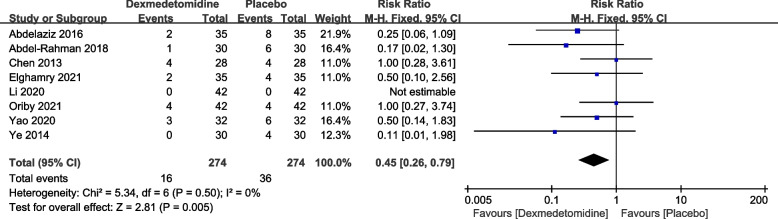
Forest plot of incidence of severe delirium DEX, dexmedetomidine

Furthermore, eight articles reported the number of cases with severe postoperative delirium. Again, no significant heterogeneity was found among the studies (*P* = 0.50, I2 = 0%). A fixed-effects model was employed. The pooled RR value was 0.45, with a 95% CI of (0.26, 0.79), and the combined effect size Z = 2.81 (*P* = 0.005). The results showed that compared to the placebo group, the dexmedetomidine group had a significantly lower incidence of severe delirium, as seen in Fig. 4. Egger's tests (*P* = 0.0063) indicated that there was no publication bias.

### Postoperative nausea and vomiting

**Fig. 5:**
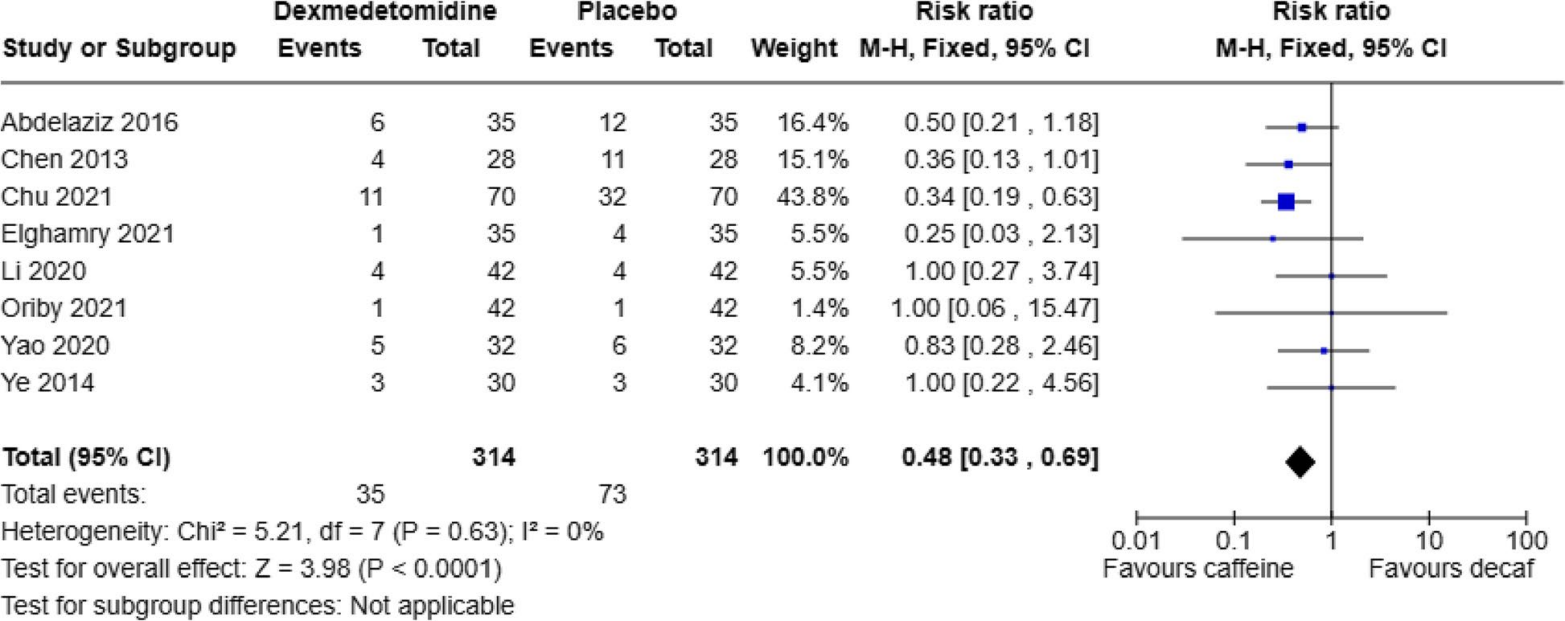
Forest plot of incidence of PONV DEX, dexmedetomidine

This study also analyzed the incidence of postoperative nausea and vomiting (PONV) with dexmedetomidine compared to a placebo (saline solution). A total of 8 articles reported the number of cases of postoperative nausea and vomiting, and no significant heterogeneity was found among the studies (*P* = 0.63, I2 = 0%). A fixed-effects model was used. The pooled RR value was 0.48, with a 95% CI of (0.33, 0.69), and the combined effect size Z = 3.98 (*P* < 0.0001). The results showed that compared to the placebo group, the dexmedetomidine group had a significantly lower incidence of PONV, as seen in Fig. 5. Egger's tests (*P* = 0.0072) indicated that there was no publication bias.

### Postoperative pain

**Fig. 6:**
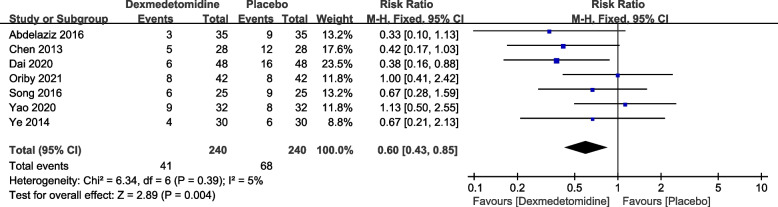
Forest plot of the number of people supplemented with analgesia DEX, dexmedetomidine

This study analyzed the incidence of the need for supplemental analgesia for postoperative pain when using dexmedetomidine compared to a placebo (saline solution). A total of 7 articles reported the number of cases requiring supplemental analgesia for postoperative pain, and no significant heterogeneity was found among the studies (*P* = 0.39, I2 = 5%). A fixed-effects model was used. The pooled RR value was 0.60, with a 95% CI of (0.43, 0.85), and the combined effect size Z = 2.89 (*P* = 0.004). The results showed that compared to the placebo group, the dexmedetomidine group had a significantly lower incidence of the need for supplemental analgesia for postoperative pain, as seen in Fig. 6. Egger's tests (*P* = 0.013) indicated that there was no publication bias.

### Oculocardiac reflex

**Fig. 7:**
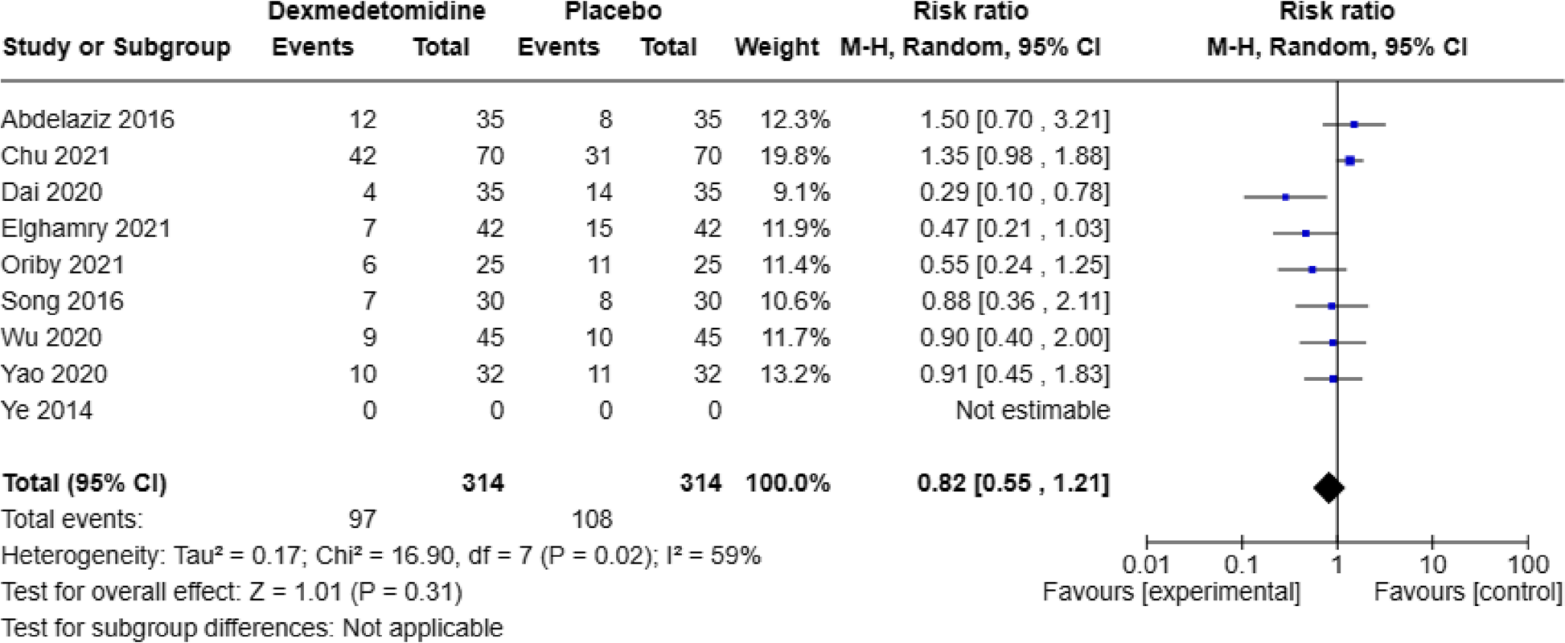
Forest plot of OCR incidence DEX, dexmedetomidine

This study analyzed the incidence of the oculocardiac reflex (OCR) when using dexmedetomidine compared to a placebo (saline solution). A total of 9 articles reported the number of cases with oculocardiac reflex postoperatively, and significant heterogeneity was found among the studies (*P* = 0.02, I2 = 59%). A random-effects model was employed. The pooled RR value was 0.82, with a 95% CI of (0.55, 1.21), and the combined effect size Z = 1.01 (*P* = 0.31). The results showed that there was no significant change in the incidence of OCR in the dexmedetomidine group compared to the placebo group, as shown in Fig. 7. Egger's tests (*P* = 0.432) indicated that there was publication bias.

**Fig. 8:**
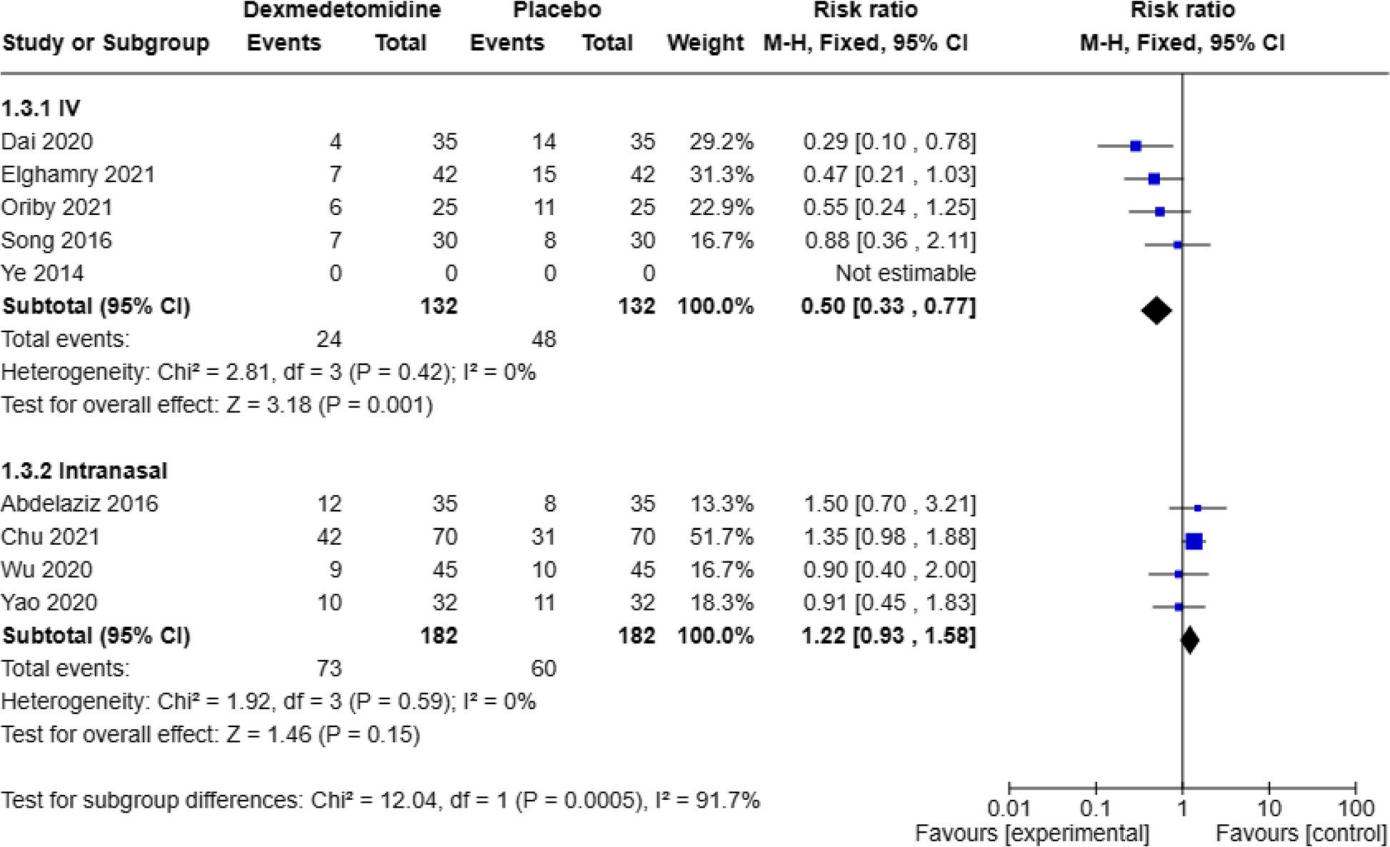
Forest plot of the subgroup of OCR incidence DEX, dexmedetomidine

Due to the significant heterogeneity and bias observed in the OCR analysis, a subgroup analysis was conducted. The studies were categorized into intravenous administration and intranasal administration groups for subgroup analysis. A total of 5 articles reported the number of cases with OCR postoperatively following intravenous administration of dexmedetomidine, and no significant heterogeneity was found among these studies (*P* = 0.42, I2 = 0%). A fixed-effects model was used. The pooled RR value was 0.50, with a 95% CI of (0.33, 0.77), and the combined effect size Z = 3.18 (*P* = 0.001). The results showed that the incidence of OCR was significantly reduced in the group receiving dexmedetomidine intravenously compared to the placebo group, as seen in Fig. 8. Egger’s tests (*P* = 0.0074) indicated that there was no publication bias. A total of 4 articles reported the number of cases with OCR postoperatively following intranasal administration of dexmedetomidine, and no significant heterogeneity was found among these studies (*P* = 0.59, I2 = 0%). A fixed-effects model was used. The pooled RR value was 1.22, with a 95% CI of (0.93, 1.58), and the combined effect size Z = 1.46 (*P* = 0.15). The results showed that there was no significant change in the incidence of OCR in the group receiving dexmedetomidine intranasally compared to the placebo group, as seen in Fig. 8. Egger’s tests (*P* = 0.0113) indicated that there was no publication bias. There was high heterogeneity between the two subgroups, with *P* = 0.0005, I2 = 91.7%.

## Discussion

This study is the most up-to-date and comprehensive systematic review and meta-analysis of RCT studies to date. In contrast to other studies that only compare the intravenous administration of dexmedetomidine [26], this study also includes the relatively new method of intranasal administration of dexmedetomidine. After searching multiple databases and screening literature, 13 studies with a total of 1018 patients were included for analysis. The meta-analysis of this study shows that the use of dexmedetomidine in strabismus surgery can significantly reduce the incidence of postoperative delirium, severe postoperative delirium, postoperative nausea and vomiting, and the need for supplemental analgesia. It was also found that intravenous dexmedetomidine can reduce the incidence of the oculocardiac reflex postoperatively.

Postoperative delirium is a common adverse complication after general anesthesia surgery, and strabismus surgery is one of the risk factors for postoperative delirium [27]. Possible risk factors for postoperative delirium include rapid awakening after anesthesia, use of short-acting volatile anesthetics, postoperative pain, age, and type of surgery [28]. Previous literature analysis found that dexmedetomidine reduced the incidence of postoperative delirium in non-cardiac surgeries [29]. However, there is still controversy regarding whether dexmedetomidine can reduce the incidence of postoperative delirium in strabismus surgery, and the results are inconsistent [17, 19]. This study did not conduct subgroup analysis for age and anesthesia methods, so the results have a certain degree of generalizability. Our results suggest that dexmedetomidine can reduce the incidence or severity of postoperative delirium. Therefore, it is recommended that anesthesiologists consider using dexmedetomidine to reduce the occurrence of postoperative delirium in patients undergoing high-risk surgeries, such as strabismus surgery.

Postoperative nausea and vomiting are common phenomena after general anesthesia and can lead to electrolyte imbalances, aspiration pneumonia, delayed discharge, and increased unplanned admissions, which significantly increase patient discomfort and medical costs [30]. There are reports that dexmedetomidine has a prophylactic antiemetic effect during general anesthesia [31], but this analysis lacks research on strabismus surgery. In this study, our results demonstrate that dexmedetomidine has a prophylactic antiemetic effect in strabismus surgery. However, there are few RCT studies comparing the antiemetic effects of dexmedetomidine with other preoperative prophylactic antiemetic drugs, and further research is needed to compare the antiemetic effects of dexmedetomidine.

Postoperative pain is the main cause of discomfort after anesthesia, and patients undergoing strabismus surgery are at high risk for postoperative pain [32]. Postoperative pain is associated with pediatric postoperative delirium, and therefore the European Society for Paediatric Anaesthesiology recommends pain management for six common pediatric surgeries, but strabismus surgery is not currently included in the guideline [33]. Dexmedetomidine has analgesic effects and is widely used in various surgeries. It can effectively relieve pain, prolong the pain-free period, and reduce the use of opioids during the recovery period after general anesthesia [34]. Our study confirms this, showing that dexmedetomidine can reduce the number of patients requiring supplemental analgesia after strabismus surgery.

Regarding other adverse events after strabismus surgery, the oculocardiac reflex is of current interest. The oculocardiac reflex is related to triggering stimuli, most commonly caused by the traction of the extraocular muscles [35]. There has been much clinical debate about the relationship between dexmedetomidine and the oculocardiac reflex, with no definitive results [36]. Interestingly, the comprehensive analysis of all the literature included in this study revealed high heterogeneity between results. Subgroup analysis by administration method showed that intravenous administration can reduce the incidence of the oculocardiac reflex, which is consistent with previous meta-analyses [26]. However, the relationship between intranasal administration and the oculocardiac reflex is still unclear, possibly due to the small number of studies on intranasal administration. More research is needed to evaluate the relationship between intranasal administration and the oculocardiac reflex.

This study has some limitations. Firstly, although the study has made efforts to include more comprehensive RCT studies, the number of studies is still small, with small sample sizes, and all studies are single-center, which may introduce bias. Secondly, there are differences in the timing and dosages of dexmedetomidine administration among different studies, but due to the small number of control experiments, intergroup analysis cannot be performed, which may affect the generalizability of the results. Additionally, postoperative management and interventions differ among studies, which may affect the heterogeneity of the results.

In summary, our study found that in patients undergoing strabismus surgery, the use of dexmedetomidine can alleviate postoperative delirium and reduce the incidence of postoperative nausea, vomiting, and pain. In addition, intravenous administration of dexmedetomidine can reduce the incidence of the oculocardiac reflex.

## Supplementary Information

**Additional file 1.**

## Data Availability

The data used and analyzed during the current study are available from the corresponding author.
